# Towards gene therapy for primary ciliary dyskinesia

**DOI:** 10.1186/2046-2530-1-S1-P109

**Published:** 2012-11-16

**Authors:** M Munye, RA Hirst, C O'Callaghan, SJ Howe, SL Hart

**Affiliations:** 1 UCL Institute of Child Health,UK; 2 University of Leicester, UK

## 

Primary ciliary dyskinesia (PCD) describes a family of rare genetic disorders affecting ciliary motility in several organ systems. The respiratory defects that can lead to lung failure, however, are most concerning. New treatments for PCD are needed that prevent progressive lung damage and we aim to develop gene therapy to achieve this. *DNAH5* is the gene most frequently mutated in PCD. It encodes a large 500kDa structural protein with ATPase activity that powers ciliary movement. PCD rarity, the shortage of genotyped patient samples and lack of suitable animal models means that better model systems are needed in which to study gene therapies. A further challenge is that gene transfer of *DNAH5* will require an efficient non-viral delivery vector capable of packaging this gene, which is too large for commonly used viral vectors. To model PCD we have used RNA interference to silence *DNAH5* in normal human bronchial epithelial cells grown in air-liquid interface cultures. Cells were transduced with a lentivirus expressing an shRNA for *DNAH5*. Silencing of *DNAH5* expression was demonstrated and preliminary evidence that the cilia were immotile. We have cloned *DNAH5* from mRNA of human ciliated cells into a mammalian expression vector and sequenced it. The *DNAH5* clone expressed both mRNA and protein in transfected cells. Transfections were performed with a nanocomplex formulation comprising liposomes and targeting peptides that we have developed for *DNAH5* gene transfer to the respiratory epithelium. We are now able to investigate PCD gene therapy using these models, *DNAH5* constructs and vector delivery system.

